# Relationship between mean daily energy intake and frequency of consumption of out-of-home meals in the UK National Diet and Nutrition Survey

**DOI:** 10.1186/s12966-017-0589-5

**Published:** 2017-09-22

**Authors:** Louis Goffe, Stephen Rushton, Martin White, Ashley Adamson, Jean Adams

**Affiliations:** 1 Institute of Health & Society, Newcastle University, Newcastle, UK; 2 Human Nutrition Research Centre, Newcastle University, Newcastle, UK; 3 Fuse – UKCRC Centre for Translational Research in Public Health, Newcastle, UK; 4 School of Natural and Environmental Sciences, Newcastle University, Newcastle, UK; 5 Centre for Diet and Activity Research, (CEDAR), MRC Epidemiology Unit, University of Cambridge, Cambridge, UK

**Keywords:** Food intake, Eating out, Out-of-home meals, Energy intake, Restaurant, Takeaway, NDNS, Food environment

## Abstract

**Background:**

Out-of-home meals have been characterised as delivering excessively large portions that can lead to high energy intake. Regular consumption is linked to weight gain and diet related diseases. Consumption of out-of-home meals is associated with socio-demographic and anthropometric factors, but the relationship between habitual consumption of such meals and mean daily energy intake has not been studied in both adults and children in the UK.

**Methods:**

We analysed adult and child data from waves 1–4 of the UK National Diet and Nutrition Survey using generalized linear modelling. We investigated whether individuals who report a higher habitual consumption of meals out in a restaurant or café, or takeaway meals at home had a higher mean daily energy intake, as estimated by a four-day food diary, whilst adjusting for key socio-demographic and anthropometric variables.

**Results:**

Adults who ate meals out at least weekly had a higher mean daily energy intake consuming 75–104 kcal more per day than those who ate these meals rarely. The equivalent figures for takeaway meals at home were 63–87 kcal. There was no association between energy intake and frequency of consumption of meals out in children. Children who ate takeaway meals at home at least weekly consumed 55–168 kcal more per day than those who ate these meals rarely. Additionally, in children, there was an interaction with socio-economic position, where greater frequency of consumption of takeaway meals was associated with higher mean daily energy intake in those from less affluent households than those from more affluent households.

**Conclusions:**

Higher habitual consumption of out-of-home meals is associated with greater mean daily energy intake in the UK. More frequent takeaway meal consumption in adults and children is associated with greater daily energy intake and this effect is greater in children from less affluent households. Interventions seeking to reduce energy content through reformulation or reduction of portion sizes in restaurants, cafés and takeaways could potentially lead to reductions in mean daily energy intake, and may reduce inequalities in health in children.

## Background

Meals purchased out-of-home are considered to be less healthy than homemade meals [1]. Out-of-home fast-food meals tend to be higher in energy, fat, salt and sugar and lower in vitamins and minerals than meals prepared at home [2–9]. In particular, meals from independent takeaway outlets are inconsistent with dietary recommendations, delivering portions that are high in energy, all macronutrients and salt [5, 8]. Whilst the mechanisms that lead to overconsumption are not fully understood [10], experiments in both laboratory and natural settings have shown that large portion sizes, particularly of energy-dense foods, contribute to the overconsumption of energy [11]. Furthermore, consumption of meals from out-of-home sources has been linked to weight gain [12, 13] and an increased risk of insulin resistance [14] and type 2 diabetes [15].

Consumption of ready-to-eat food from out-of-home outlets such as cafés, takeaways, restaurants, and convenience stores is common [16, 17]. Our previous work identified that during 2008 to 2012 in the UK almost one-quarter of adults and one-fifth of children ate a meal out weekly and one-fifth of both adults and children ate a takeaway meal at home at least weekly [18]. A comparable study in the United States, during 2007 to 2010, found that adults consumed on average 11.3% of their energy intake from fast-food [19]. The proportion of household budgets spent on out-of-home meals has increased. In the United States in the 1970s approximately 20% of food expenditure was spent on out-of-home food, [20] rising to 38% by 1992 [17] and 50.1% in 2014 [21]. In the UK, excluding alcohol, the proportion of household food and drink budgets spent out-of-home has risen from 21% in 1995 to 26% in 2014 [22]. This increase in expenditure corresponds to an increase in availability [23], particularly with regards to multinational chain restaurants, which have pursued rapid global expansion since the 1980s [24].

Increased intake of takeaway food is associated with increased exposure to takeaway outlets [25]. A number of epidemiological studies have detailed the association between increased exposure and weight gain, obesity, insulin resistance and type 2 diabetes [12, 14, 15]. The consumption of out-of-home meals is socio-demographically patterned; our analysis of UK data found that boys eat takeaway meals more frequently than girls and peak consumption is found in young adults between the ages of 19 to 29 [18]. We also found that more affluent adults were more likely to eat meals out at least once per week, but children from less affluent households were more likely to eat takeaway meals at least weekly [18]. Additionally, exposure to out-of-home food outlets is strongly socio-demographically patterned, since there is greater takeaway outlet density in more deprived communities [23, 26–28].

As a result of these emerging associations, there have been a number of studies in the US that have attempted to quantify the effect of food from fast-food and full-service restaurants on dietary intake whilst adjusting for socio-demographic variables [29–32]. In adults, consumption of any food from a fast-food outlets or full-service restaurants was respectively associated with a 190 and 187 kcal greater daily energy intake [30]. The equivalent figures for children (aged 2 to 11 years) were an additional 126 and 160 daily kilocalories [29] and adolescents (aged 12 to 19 years) were 310 and 267 kcal [29]. However, to our knowledge, no work has been carried out on UK populations exploring how habitual patterns of out-of-home meal consumption impact on mean daily energy intake across all ages.

We hypothesised that mean daily energy intake is dependent on frequency of consumption of out-of-home meals in both adults and children (hypothesis 1). We also hypothesised that this relationship is dependent on age, gender, socio-economic position and body size (hypothesis 2).

## Methods

We undertook secondary analysis of individual-level data from a large, annual UK cross-sectional survey of adults and children to estimate the impact on mean daily energy intake of frequency out-of-home meal consumption, including both meals eaten out and takeaway meals eaten at home, whilst adjusting for a range of socio-demographic and anthropometric factors.

### Data source

We combined data from the first four annual waves of the UK National Diet and Nutrition Survey (NDNS) from 2008 to 9 to 2012–13. The NDNS is a rolling programme of cross-sectional surveys carried out across the United Kingdom. NDNS aims to recruit 1000 individuals per year, 500 adults aged 19 years and over, and 500 children aged 1.5 to 18 years, broadly representing the UK population, and collects data on food consumption, nutrient intake and nutritional status of people living in private households. As far as possible sampling, recruitment and data collection methods are constant across years to enable data to be combined across survey years [33]. Individuals in the study completed an estimated four-day food diary and participated in an interview to collect background data that included data on dietary habits, socio-demographic status and lifestyle [33]. Overall, 91% of households eligible for inclusion agreed to take part in the first four waves of NDNS. Usable food diaries (three or four completed days) were collected from at least one household member in 58% of eligible households. At an individual level, 56% of those selected to take part completed usable food diaries: 2083 adults and 2073 children [33].

### Variables

#### Mean daily energy intake

For each individual, mean daily energy intake (kilocalories) was derived from food diary data. The NDNS does not collect data on the source of the food consumed.

#### Socio-demographic variables

We included three socio-demographic variables to test the second hypothesis: age (years), gender (male/female) and socio-economic position (SEP). SEP was measured using the National Statistics Socio-economic Classification (NS-SeC) [34], where individuals are assigned a class based on the employment of the person in their household with the highest income. Data were collapsed into two levels for analysis – Class 1 (managerial, professional and intermediate occupations) and Class 2 (routine and manual occupations) – as this maximised model fit, as measured by Akaike Information Criterion (AIC). Individuals were excluded where either a response was not provided or the householder had never worked. We anticipated that SEP will impact on behaviour but not necessarily in a monotonic way (e.g. increasing or decreasing with SEP). Therefore, we used SEP as a grouping variable to analyse the differences between classes.

#### Anthropometric variables

There are many ways of quantifying body size. We considered both body mass index (BMI) and the cubic transformation of height. The cubic transformation of height provides a body mass measure that does not include an estimate of specific tissue mass (e.g. muscle, fat or bone). In full model analysis the cubic transformation of height variable resulted in better model fit than BMI, as measured by AIC.

#### Frequency of eating meals out and takeaway meals at home

The NDNS does not contain details about where food reported in food diaries was obtained but the interview contained two questions to estimate habitual out-of-home food consumption. These were: “On average, how often do you/does [child’s name] eat meals out in a restaurant or café?”; and “On average, how often do you/does [child’s name] eat takeaway meals at home?”. Individuals were informed by the researcher leading the interview that “‘meals means more than a beverage or bag of chips”. The responses available to individuals fell on a five point ordinal scale: “Rarely or never?”; “1–2 times per month”; “1–2 times per week”; “3–4 times per week”; or “5 or more times per week”. Due to the low number of individuals reporting frequency of consumption in the highest three categories, these were merged to form one category “1 or more times per week”.

### Data analysis

We used generalized linear modelling (GLM) to investigate the relationships between frequency of consumption of out-of-home meals and individual’s mean daily energy intake, with separate models for adults (aged 19 years and over), and children (aged 1.5 to 18 years) (Hypothesis 1). To explore the influence of socio-demographic variables, we included gender, age, height cubed, NS-SeC, frequency of meals out and frequency of takeaway meals as independent predictors (Hypothesis 2). In the adult model, we included potential interactions between: age and both frequency of meals out and takeaways (as consumption peaks in young adults [18]); and NS-SeC and meals out (as adults living in more affluent household are more likely to eat meals out at least once per week [18]). In the child model we included potential interactions between: gender and takeaway meal consumption (as more boys than girls eat takeaway meals [18]); age and both frequency of meals out and takeaways (as consumption peaks in young adults [18]); and NS-SeC and takeaway meal consumption (as children living in less affluent households are more likely to eat takeaways [18]). We used stepwise deletion to identify the significant independent variables (*P* < 0.05). Using the derived regression equations from the GLMs we were able to estimate mean daily energy intake for both adults and children, adjusted for key socio-demographic and anthropometric variables to illustrate the relationship with habitual consumption of out-of-home meals.

In order to illustrate the relationship between mean daily energy intake and the frequency of consumption variables in both adults and children, the derived regression equations from the GLMs were used to generate estimates, which were transformed into kilocalories and plotted with errors bars representing 95% confidence intervals. To calculate the estimates for adults we used the mean height for both females and males in the modelled dataset, 161 cm and 175 cm respectively. To calculate the estimates for the children we used the mean height for both females and males in the modelled dataset, 138 cm and 139 cm respectively.

We carried out data analyses in R [35].

## Results

We only included individuals in the analysis where there was a complete set of records for the variables of interest. This resulted in 1889 (90.7%) adults and 1797 (86.7%) children from the first four annual waves of the NDNS (2008–9 to 2012–13). A summary of variables of interest for adults is shown in Table 1 and for children in Table 2. Since the data were not complete for all individuals, some individuals were omitted from the analyses. We assessed the extent to which excluded and included cases differed in their mean daily energy intake, socio-demographic, anthropometric characteristics and frequency of consumption of out-of-home meals variables using chi-squared tests and GLMs, as appropriate. Significant differences in the adult model included: mean daily energy intake, where included cases were more likely to have a higher mean daily energy intake; NS-SeC, where included cases were more likely to be in a higher social group; and frequency of meals out consumed, where included cases were more likely to eat meals out more frequently. Significant differences in the child model included: mean daily energy intake, where included cases were more likely to have a higher mean daily energy intake; age, where included cases were more likely to be older; frequency of meals out consumed, where included cases were more likely to eat meals out more frequently; and frequency of takeaway meals consumed, where included cases were more likely to eat takeaway meals more frequently.

**Table 1:** Summary of adult model variables

Variable	Level	N (%)	Mean (SD)
Categorical variables			
All adults		1889	
Gender	Male	829 (43.9)	
	Female	1060 (56.1)	
NS-SeC	Class 1: Higher managerial, administrative, professional and intermediate occupations	1217 (64.4)	
	Class 2: Routine and manual occupations	672 (35.6)	
Frequency of eating meals out	Rarely or never	537 (28.4)	
	1–2 times per month	851 (45.1)	
	1 or more times per week	501 (26.5)	
Frequency of eating takeaway meals at home	Rarely or never	848 (44.9)	
	1–2 times per month	667 (35.3)	
	1 or more times per week	374 (19.8)	
Continuous variables			
Age	Years		49.2 (16.9)
Height	Centimetres		167.5 (9.5)
Mean daily energy intake	Kilocalories		1811.2 (573.1)

**Table 2:** Summary of child model variables

Variable	Level	N (%)	Mean (SD)
Categorical variables			
All children		1797	
Gender	Male	934 (52.0)	
	Female	863 (48.0)	
NS-SeC	Class 1: Higher managerial, administrative, professional and intermediate occupations	1158 (64.4)	
	Class 2: Routine and manual occupations	639 (35.6)	
Frequency of eating meals out	Rarely or never	497 (27.7)	
	1–2 times per month	923 (51.4)	
	1 or more times per week	377 (21.0)	
Frequency of eating takeaway meals at home	Rarely or never	682 (38.0)	
	1–2 times per month	733 (40.8)	
	1 or more times per week	382 (21.3)	
Continuous variables			
Age	Years		9.8 (5.0)
Height	Centimetres		138.4 (27.8)
Mean daily energy intake	Kilocalories		1595.3 (462.1)

### Adults

Adult mean daily energy intake was dependent on gender, body size, NS-SeC, frequency of eating meals out, and in the highest consumers of takeaway meals (Table 3).

**Table 3:** Significant coefficients from adult GLM

Coefficients	Estimate (Std. Error)	t value	Pr(>|t|)
Intercept	6.9156 (0.0507)	136.4009	< 0.001
Gender: Male	0.1733 (0.0188)	9.2008	< 0.001
Body size	< 0.001 (< 0.001)	7.6875	< 0.001
NS-SeC: Class 2	−0.0457 (0.0139)	−3.2976	< 0.001
Frequency of eating meals out: 1–2 times per month	0.0518 (0.0159)	3.2622	0.0011
Frequency of eating meals out: 1 or more times per week	0.0526 (0.0180)	2.9124	0.0036
Frequency of eating takeaway meals at home: 1–2 times per month	0.0256 (0.0149)	1.7243	0.0848
Frequency of eating takeaway meals at home: 1 or more times per week	0.0442 (0.0179)	2.4754	0.0134

AIC: 586.61

Null deviance: 194.80 on 1888 degrees of freedom

Residual deviance: 149.45 on 1881 degrees of freedom

D-squared: 0.23

Men consumed more energy per day (*t* = 9.20, *P* < 0.01), as did larger adults (*t* = 7.69, *P* < 0.01) and those living in routine and manual households consumed less (*t* = −3.30, *P* < 0.01). Adults who ate out more frequently consumed more energy, 1–2 times per month (*t* = 3.26, *P* < 0.01), 1 or more times per week (*t* = 2.91, *P* < 0.01). Only adults who ate takeaway meals most frequently (1 or more times per week (*t* = 2.48, *P* = 0.01)), consumed significantly more energy. There was a suggestion of a positive effect of eating takeaway meals at home 1–2 times per month, but this was not significant (*t* = 1.72, *P* = 0.08). There were no significant interaction terms in the adult GLM relating to mean daily energy intake.

### Children

Child mean daily energy intake was dependent on gender, body size and NS-SeC (Table 4).

**Table 4:** Significant coefficients from child GLM

Coefficients	Estimate (Std. Error)	t value	Pr(>|t|)
Intercept	7.0228 (0.0156)	449.9743	< 0.001
Male	0.1114 (0.0116)	9.5896	< 0.001
Body size	< 0.001 (< 0.001)	21.3252	< 0.001
NS-SeC Class 2	−0.0449 (0.0203)	−2.2084	0.0273
Eating takeaway meals 1–2 times per month	0.0267 (0.0162)	1.6471	0.0997
Eating takeaway meals 1 or more times per week	0.0392 (0.0205)	1.9083	0.0565
Interaction: NS-SeC Class 2: Eating takeaway meals 1–2 times per month	0.0558 (0.0278)	2.0076	0.0448
Interaction: NS-SeC Class 2: Eating takeaway meals 1 or more times per week	0.0682 (0.0325)	2.1020	0.0357

AIC: 60.33

Null deviance: 148.00 on 1796 degrees of freedom

Residual deviance: 107.72 on 1789 degrees of freedom

D-squared: 0.27

Boys consumed more (*t* = 9.56, *P* < 0.01) as did larger children (*t* = 21.33, *P* < 0.01) and those living in routine and manual households consumed less (*t* = −2.21, *P* = 0.03). There was a suggestion that children who ate takeaway meals more frequently consumed more: 1–2 times per month (*t* = 1.65, *P* < 0.10), 1 or more times per week (*t* = 1.91, *P* = 0.06). Despite this non-significance, the frequency of consumption of takeaway meals at home variables were retained due to a significant interaction between NS-SeC and frequency of eating takeaway meals at home, where the model estimate increased as frequency of consumption increased, 1–2 times per month (*t* = 2.01, *P* = 0.04), 1 or more times per week (*t* = 2.10, *P* = 0.04). This interaction represents a synergistic effect of the two single dependent variables alone. The mean daily energy intake of children living in routine or manual households was more positively related to a greater frequency of consumption of takeaway meals at home than in children living in higher managerial, administrative, professional and intermediate households.

### Model residuals

For a GLM with a Gaussian error structure to be an adequate model for the data, the residuals (error) should be normally distributed with zero mean. The distribution of residuals from the adult and child models were normal but there were outliers at the lower end of the body size (height cubed) range where the model appeared to overestimate. Consideration of the records for the individuals concerned showed a level of recorded mean daily energy intake that was lower than the intake required to maintain an estimated basal metabolic rate, indicating poor or inadequate recording.

**Fig. 1:**
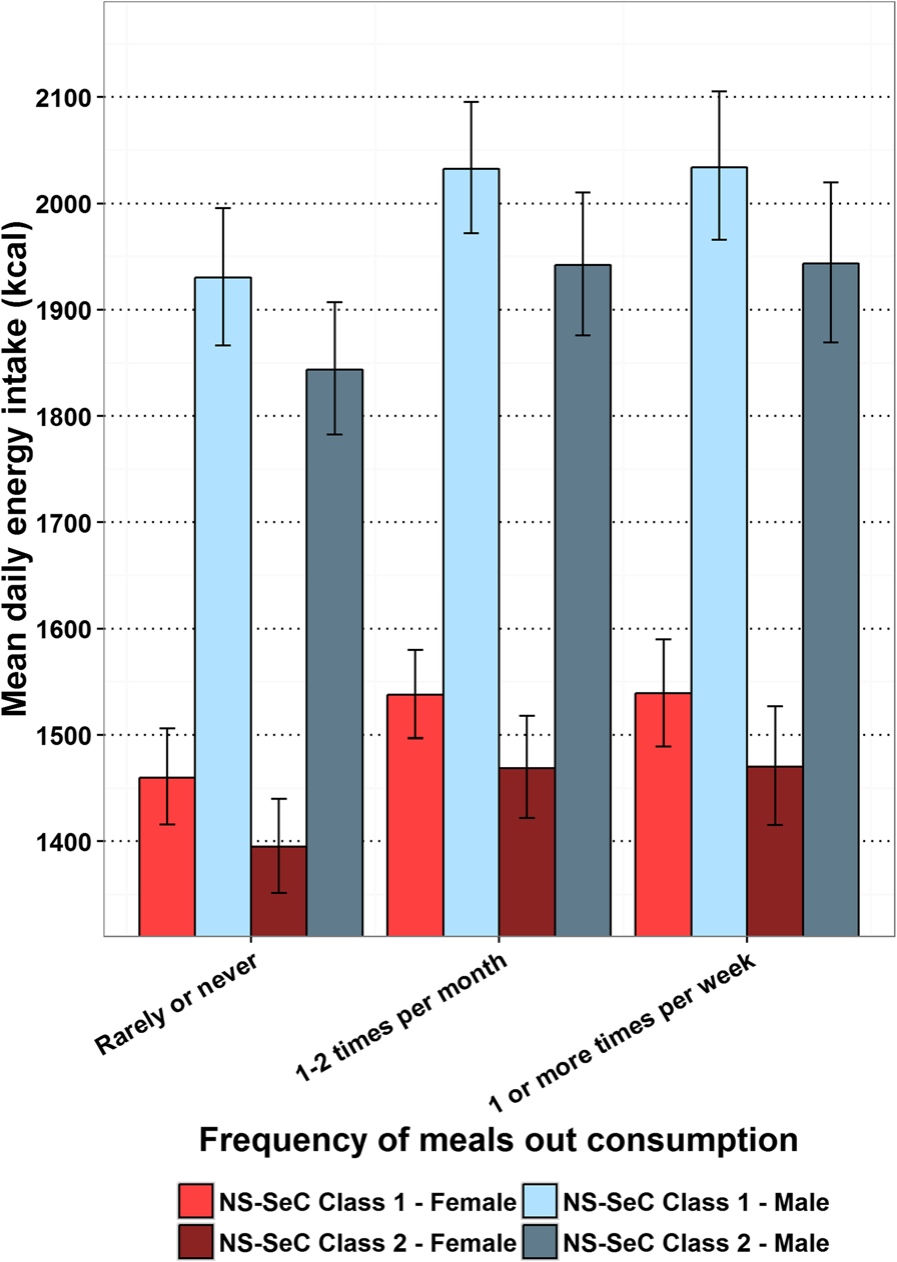
Estimated adult mean daily energy intake by frequency of meals out consumption with error bars representing 95% confidence intervals

**Fig. 2:**
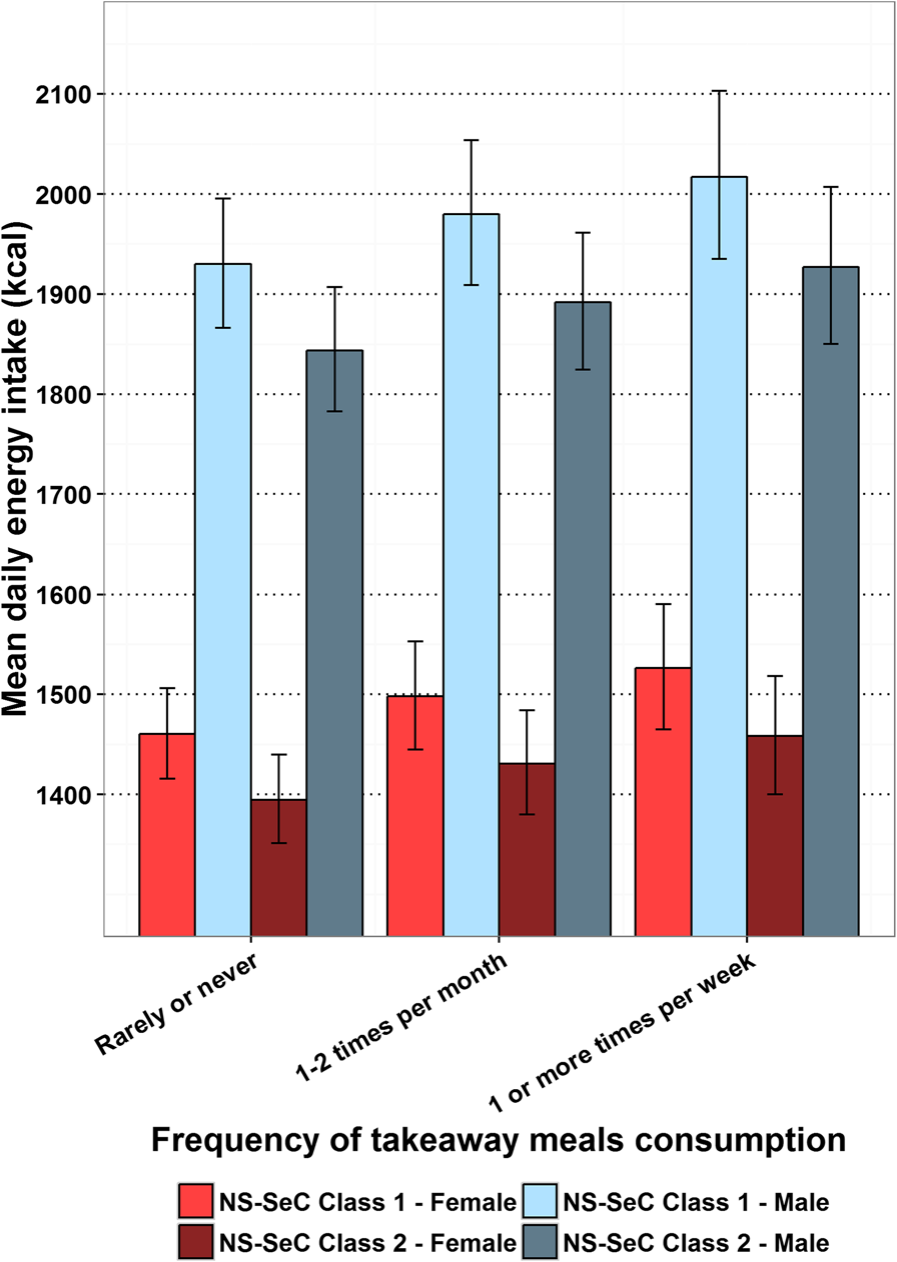
Estimated adult mean daily energy intake by frequency of takeaway meal consumption with error bars representing 95% confidence intervals

**Fig. 3:**
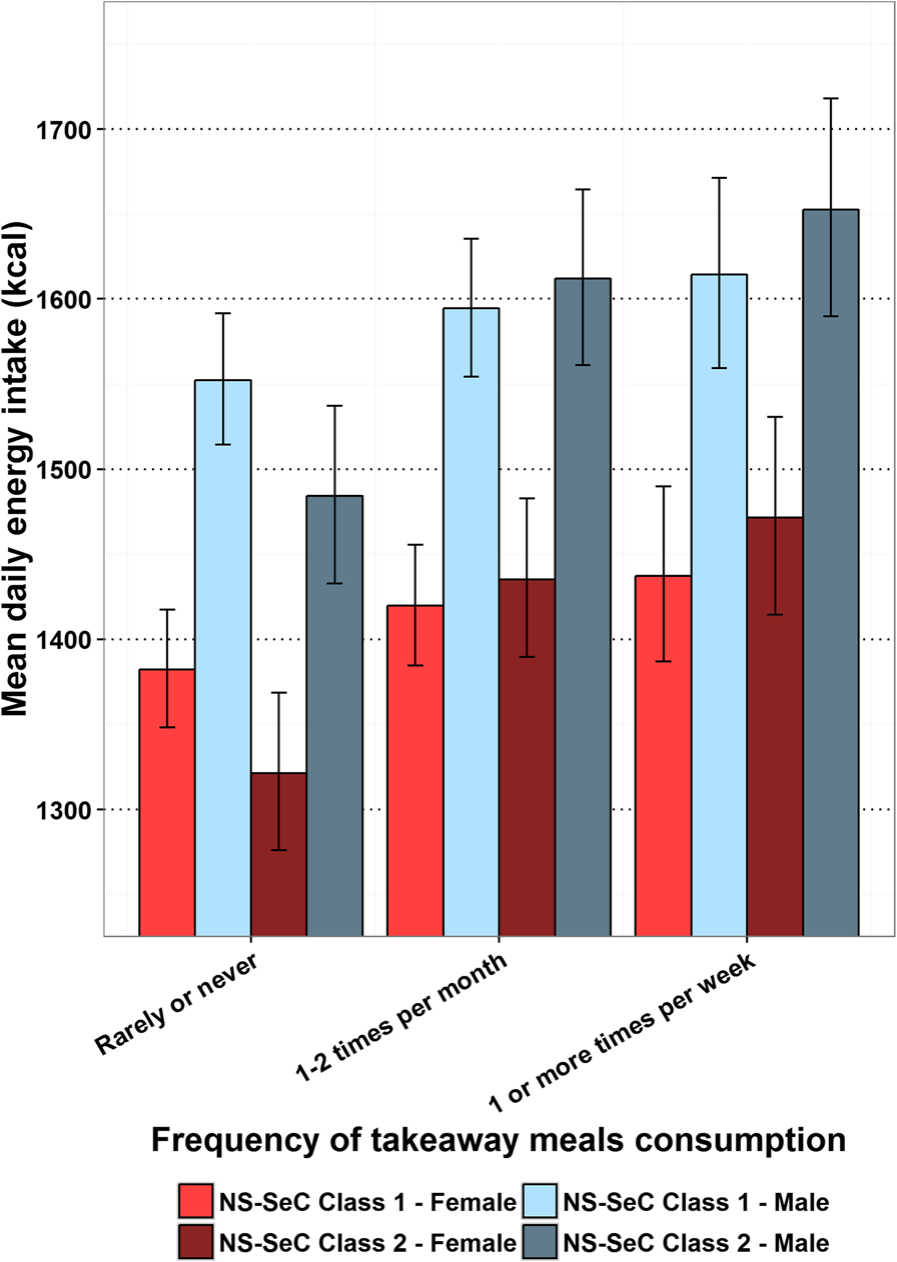
Estimated child mean daily energy intake by frequency of takeaway meal consumption with error bars representing 95% confidence intervals

The estimated mean daily energy intake in adults from the GLMs in relation to the frequency of meals out consumed are shown in Fig. 1. This suggests there is an upward trend between a greater consumption of meals out and mean daily energy intake, which levels off at a consumption of 1–2 meals out per month in all groups modelled. The estimated mean daily energy intake in adults from the GLMs in relation to the frequency of takeaway meals consumed is shown in Fig. 2, which suggests greater consumption of takeaway meals is associated with greater mean daily energy intake in all groups modelled. The estimated mean daily energy intake in children from the GLMs in relation to the frequency of takeaway meals consumed are shown in Fig. 3. Here there is also a trend suggesting greater consumption of takeaway meals is associated with greater mean daily energy intake. However, this association is more marked in those individuals from NS-SeC Class 2. There is no figure illustrating the impact of frequency of meals out on mean daily energy intake in children as this variable was non-significant in the child GLM. The estimated difference in mean daily energy intake between the highest and lowest consumers of both meals out and takeaway meals are shown in Table 5.

**Table 5:** Estimated difference in mean daily energy intake between the highest and lowest consumers

		Difference in consumption between ‘Rarely or never’ and ‘1 or more times per week’	
		Meals out (kcal)	Takeaways (kcal)
Adult	NS-SeC Class 1 - Male	104	87
	NS-SeC Class 2 - Male	100	83
	NS-SeC Class 1 - Female	79	66
	NS-SeC Class 2 - Female	75	63
Child	NS-SeC Class 1 - Male		62
	NS-SeC Class 2 - Male		168
	NS-SeC Class 1 - Female		55
	NS-SeC Class 2 - Female		150

## Discussion

### Summary of principal findings

We found a positive relationship between habitual consumption of out-of-home meals and mean daily energy intake. In adults, after adjusting for key socio-demographic and body size variables, we found that greater habitual frequency of consumption of both meals out in a restaurant or café, and takeaway meals was associated with greater mean daily energy intake. Adults who ate meals out at least weekly consumed on average 75–104 kcal more per day than those who ate these meals rarely. Comparable figures for eating takeaway meals at home at least weekly were 63–87 kcal. In children, only habitual consumption of takeaway meals at home had a suggested positive relationship with mean daily energy intake. Children who ate takeaway meals at home at least weekly consumed 55–168 kcal more than those eating these meals rarely. In addition, we found that the impact was amplified by SEP in children, where the larger mean daily energy intake associated with habitual consumption of takeaway meals at home was greater in children from less versus more affluent households.

### Strengths and limitations of study

The modelling method that we applied allowed us to investigate a range of explanatory variables. The range of significant relationships we found indicates that our analyses are unlikely to be underpowered. Although the NDNS attempts to attain a nationally representative sample, because some records were excluded from our analyses due to incomplete data, our results may not be generalisable across the UK. However, it is unclear why the relationships we have identified might vary in other UK groups. As the UK out-of-home food environment is unique [36, 37], our findings may not apply to settings outside the UK. The NDNS does not publish data on participants who did not complete a food diary. Therefore, there is no straightforward method to compare characteristics between those who completed a food diary and those who did not. The NDNS acknowledges that non-response bias exists in their sample and provide survey weights. While there are advantages of using weights for simple population averages, it is not clear how such weights are applied to more complex methods (e.g. regression coefficients). Creating weights requires arbitrary choices regarding inclusion of weighting factors and interactions. We chose not to use these weights in our analysis. However, each of our models were appropriately adjusted for by model covariates in order to take account of potential confounding by socio-demographic variables. While there were some individuals that had recorded mean daily energy intake that was lower than the intake required to maintain an estimated basal metabolic rate, indicating potentially aberrant data, we cannot state conclusively if they were erroneous records. Therefore, no data was excluded from our analysis.

A limitation of the data is that the two self-reported out-of-home consumption variables have not been validated and do not detail what out-of-home food was purchased. If systematic variation exists between what different socio-demographic groups purchase in terms of out-of-home meals and what types of outlets they frequent, this could have led to bias. A related study in Irish children cautioned that assessment of out-of-home food intake using questionnaire data might lead to underreporting, specifically when compared to food diary data [38]. Therefore, the two out-of-home consumption variables that we used may underestimate actual consumption. Daily energy intake data based on food diary data is also prone to misreporting, particularly underreporting, and there is some evidence this varies by age [39] and BMI [40].

We used a binary variable measure of SEP based on NS-SeC, which produced a better fitting GLM than the original eight class equivalent, as measured by AIC. Other SEP measures are available in NDNS including markers of education and income. We did not use these as the majority of child participants were still in full time education and a large proportion of participants refused to give details regarding their income.

Our data were cross-sectional and as such we cannot conclude that there is a definitive causal relationship between habitual consumption of meals out or takeaway meals at home and mean daily energy intake, nor the direction of causation between these variables.

### Comparison with other studies and interpretation of findings

Burgoine et al. (2014) showed that exposure to takeaway food outlets was positively associated with both increased consumption of out-of-home foods and with a higher BMI and obesity in UK adults [25]. Our work provides a potential explanation as to why the increased consumption may lead to an increased BMI and obesity through individuals increasing their overall mean daily energy intake as a result of increased habitual consumption of out-of-home food.

Our results reflect a previous systematic review which found that eating out-of-home was associated with a higher daily energy intake [1]. Six of the ten studies included in this systematic review used data that was both from a Western country and that was either nationally representative or from a large cohort. Of these comparable studies only one found no significant influence of food consumed out-of-home on daily energy intake - in Irish children aged 5–12 [38]. The difference between studies could potentially be explained by contextual differences in out-of-home environments, differences in populations studied (we included participants aged 1.5 years and older, rather than just 5–12 years) or details of data used. Whilst the Irish study used data on where all food eaten over a short period was prepared or obtained, we measured habitual consumption of out-of-home food over the longer term.

Comparable modelling studies that explored the association between fast-food consumption and diet quality in the US [29, 30, 32] also found significant positive associations between frequency of fast-food consumption and energy intake. In US adults, consumption of any fast-food or full-service restaurant meals on a given day was associated with a daily energy intake increase of 190 and 187 kcal respectively [30], in children (aged 2 to 11) an increase of 126 and 160 kcal [29] and adolescents (aged 12 to 19) an increase of 310 and 267 kcal [29]. Our models estimated a mean daily difference of 63–87 kcal in adults and 55–168 kcal in children eating takeaway meals at least weekly compared to rarely, and 75–104 kcal per day in adults eating meals out at least weekly compared to rarely. Although our findings are not directly comparable, both studies found a positive association between greater out-of-home meal consumption and daily energy intake. Our estimates represent a sizeable difference in mean daily energy intake in comparison to government dietary recommendations. The UK government recommends that both adult and 15 to 18 year old females should consume 2000 kcal per day [41]. Therefore, adults and children in our study who ate takeaway meals at least weekly are respectively expected to consume 3.2%–4.4% and 2.8%–8.4% more energy per day than those that consume takeaway meals rarely. Comparable figures for eating meals out at least weekly in adults are 3.8%–5.2%. In adults, the mean daily energy intake estimate for eating meals out was greater than for takeaways. This may be due to cultural practices, such as when eating out is linked to celebrating specific events leading to a combination of consumption of multiple courses and beverages.

Of particular interest in our study is the relationship between takeaway meal consumption and SEP in children, suggesting an amplified impact of takeaway consumption on mean daily energy intake in children from a lower SEP. This combined with the established relationship between deprivation and the density of takeaway outlets [23, 26–28], means that children living in less affluent areas may be both more exposed to and more susceptible to the effects of eating takeaway meals. An also reported that daily energy intake appeared larger among individuals from lower SEP [30], and a UK study that found that greater fast-food consumption, BMI, and odds of obesity were associated with greater fast-food outlet exposure and lower SEP [42], also suggesting amplification of a neighbourhood effect on inequalities in diet and obesity.

### Implications for policy and practice

Our results suggest that increased frequency of consumption of meals out in a restaurant or café by adults, and takeaway meals at home by both adults and children is likely to be associated with an increase in mean daily energy intake. With a secular trend towards increasing exposure and ease of access to out-of-home food outlets through online portals [43], and increased expenditure [21, 22], this has potential to have an adverse impact on overall diet quality. Policy makers, local government and caterers should therefore consider options that aim to limit consumption of such meals, or seek to improve the nutritional quality of out-of-home food, primarily by reformulating, reducing portion size and by providing customers with suitable information to enable them to make informed choices [44]. However, to date there is limited evidence regarding the effectiveness of such interventions [45, 46].

We found the association between habitual consumption of takeaway food and mean daily energy intake to be greater in children from less versus more affluent households. Addressing known socio-economic differences in neighbourhood exposure to takeaway outlets, and possible socio-economic differences in nutritional composition of food chosen, may help reduce known socio-economic differences in diet and obesity in children.

### Unanswered questions and future research

The nutritional profile of out-of-home food meals varies greatly [5] and consumption varies by socio-demographic group [18]. But we do not know if there is any socio-demographic patterning in the type of out-of-home food consumed – and this may explain the interaction between SEP and takeaway consumption on mean daily energy intake we found in children. Furthermore, it is not clear if out-of-home food is consumed in total by the purchaser, shared with others, or wasted – or any determinants of this. Future work could explore these points further in order to help tailor and target different interventions to different outlets and socio-demographic groups [18].

This study adds to the substantial body of evidence that suggests that frequent out-of-home food consumption is not conducive to health [1–6, 8, 14, 15, 19, 25, 30, 32, 36]. There remains limited evidence concerning what interventions might be effective in this area [45, 46] but the needs of all stakeholders, including out-of-home food vendors, [47] need to be taken into account in developing intervention strategies. Future work should focus on interventions that aim to reduce the portion size and energy density of meals in cafés, restaurants and takeaways, but also help customers to make healthier choices and incentivise outlet vendors to provide an increased range of healthy options.

## Conclusions

Using data from a large UK cross-sectional study we modelled and estimated the impact on mean daily energy intake of habitual consumption of meals eaten out in a restaurant or café and takeaway meals eaten at home in both adults and children, whilst adjusting for socio-demographic and anthropometric measures. In adults, at least weekly consumption of meals out in a restaurant or café was associated with consuming 75–104 kcal more per day compared to rarely eating these meals; and at least weekly consumption of takeaways meals at home was associated with consuming 63–87 kcal more per day. In children, only consumption of takeaway meals at home had a positive association with mean daily energy intake; with at least weekly consumption associated with consuming 55–168 kcal more per day. Additionally, the impact of consumption of takeaway food was amplified in children from less affluent households, suggesting that children of such households are more susceptible to the effects of consumption from takeaway meals than those from more affluent households. Future work should identify interventions that seek to redress the positive association between consumption of out-of-home food and daily energy intake.

## Abbreviations

AIC: Akaike information criterion
BMI: Body mass index
GLM: Generalized linear models
kcal: Kilocalories
NS-SeC: National statistics socio-economic classification
SEP: Socio-economic position
